# Cases of Dyspepsia Successfully Treated on the Purgative Plan

**Published:** 1844-03

**Authors:** William M. Yandell

**Affiliations:** Benton, Mississippi; Louisville


Art. II.—*Cases of Dyspepsia successfully treated on the purgative plan.* By William M. Yandell, M.D., of Benton, Mississippi.

The cases of Dyspepsia about to be described are clearly referrible to a miasmatic origin. In this form of the complaint, the liver is disordered, frequently enlarged, generally more or less tender to the touch. The secretion of the stomach is deficient or perverted. It follows badly cured cases of fever, or attacks persons living in malarious districts. Diet in this form of dyspepsia is of little avail. Exercise moderates the symptoms, but does not subdue them. Tonics are mischievous. The countenance of the patient is sallow; his appetite is often good, or even excessive. He has headache, constipation, furred tongue, cardialgia, peevish temper, or gloomy; and as it advances, the heart becomes troubled with palpitations, and the nervous system falls into paralysis: or if there be a predisposition to tubercles, the patient falls a victim to pulmonary consumption.

Case I.—Mrs. A., aged about 23 years, was brought to my house in February, 1839, in a carriage from a distance of 40 miles. She had been confined to her room for four years, most of the time unable to walk across the room without assistance. Her symptoms were as follows: skin very sallow, dry and constricted, with alternate flushings and chilliness, burning in the hands and feet, pain in the head, eyes of a muddy red complexion, with an unnatural fierceness; wakefulness; a dry cough; pain in the breast, low down, and extending round to the scapula; variable appetite, and all substances taken, even cold water gave pain; palpitation of the heart; constipation; pain in the region of the kidneys.

I thought I saw in these symptoms a call for the use of remedies that would promote the secretion of the liver; accordingly I gave her 40 grains of calomel. In the morning, no action from it. Gave her six pills of scammony, aloes and rhubarb. The effect was two scanty, rather dark colored discharges.

The night following, I gave her 80 grains of calomel, and in the morning eight pills of the above composition. Discharges were five or six in number, consistent and of dark color. This plan was kept up—calomel at night, and the pills in the morning—for eight days, at the end of which time her mouth had become slightly sore. The calomel was then laid aside, and the pills continued, which maintained the action of the bowels, but not so freely. Her gums were well in a few days, and she was put upon the use of pills of cal., al. and rhu., each two grains, every night, to be taken in doses of from six to twelve. This course was persevered in for seven weeks, the pills being repeated in the morning, if they failed to act sufficiently. At the end of this period, her discharges had become natural, her digestion good, and her strength greatly improved, insomuch that she was able to ride home on horseback in more comfort than she travelled to my house in a carriage. In two weeks after she began to take the pills her rest at night became comfortable. In this case I tried tonics, but found them to aggravate all the symptoms. Coffee was one of the most disturbing articles that she could use. Besides the pills, she took carb. sod. or potas. when troubled with acidity; and except this, she relied upon the pills alone. She returned home in seven weeks, with pills to be taken *pro re nata*, and under their use continued to improve daily. I saw her in the fall, seven months afterwards, and she was then enjoying excellent health, with the fresh complexion of a girl, and weighing 140 pounds.

Case II.—I was requested to visit Mrs. L., aged about 22, in March, 1839. Her health had been declining for a year or two, about which time she had an attack of bilious fever. I found her greatly depressed in spirits, complaining of vertigo and head-ache, palpitation of the heart, acidity, sleeplessness, dry cough, pain in the lower part of the chest, extending towards the scapula; enlarged spleen; dry skin, of sallow appearance. At times she felt pain in the region of the kidneys. Her tongue was slightly coated, bowels costive, or the reverse.

My first prescription was calmel 25 grains, to be followed next morning with pills of al., scam. and rhu. She objected to both remedies, alleging that calomel always salivated her, and that she could not swallow pills. I explained my purpose in administering these medicines to be, to bring away dark-colored passages, and assured her that with this result, she would find herself greatly relieved. Finally she consented, and the calomel was taken at night, followed by powders of the same ingredients as the pills next morning. As she feared, a slight soreness of her gums was the consequence of taking the calomel. She persevered in the use of the powders for some time with manifest advantage: but they were so disagreeable that she discontinued taking them too soon, and the consequence was a return of the most distressing symptoms, particularly of the pain in the lumbar region. Another physician was called to her in my absence, who pronounced her case a disease of the uterus, for which he put her upon a course of medicine. Having given the remedies for the supposed uterine disease as full a trial as the physician himself deemed necessary, and having continued to decline until her friends gave her up to die, her husband prevailed on her to return once more to the use of the powders. They brought off dark discharges, and she improved in health, until she was able to walk about the house, and in this state passed through the winter. As warm weather returned, she began to decline again, and I was once more requested to visit her. My prescription was the same as at my first visit: to-wit, 30 grains calomel, to be worked off by the powders. The result was, as in the first instance, a slight ptyalism, but much subsequent relief, as she voided a large quantity of dark, vitiated matter from the bowels.

She did not long persevere, however, in the use of the powders, the dose necessary to act being large, and the medicine exceedingly nauseous to her; and when she discontinued, the symptoms of her old malady reappeared. In a short time after abandoning them, she was confined to her bed, and became so much emaciated that her weight was only 68 pounds. For six weeks she was not able even to turn herself in bed. After returning to the powders again, which she did as the last resource, she became able to sit up in bed, in the evening, after her bowels had been moved in the morning; for she entered upon the use of the purgative when her debility was at its height. The purgative plan was kept up for three months. When the powders failed to act, I gave her a little calomel or blue pill to excite the liver gently, and I was interested to observe that the oftener the mercurial was given, the slighter was the ptyalism induced by it, until this effect ceased altogether. At the end of three months, she was able to leave her room, and by an occasional dose of the powders through the winter, her health progressively improved. She is now (January, 1842), in excellent health, having increased in weight from 68 to 118 pounds.

In this case, I tried tonics and stimulants, but found that they invariably aggravated the symptoms. Blisters were also tried, but with no better results. For six weeks the patient lived on gruel alone. Carbonate of soda acted benignly when acid was present in the stomach, relieving the flatulence which was more troublesome than ever I saw it. The alkalis are valuable remedies for this indication, and harmonize well with the purgatives.

I give calomel more freely in dyspepsia than is recommended by authors, and I have found it a most efficient remedy. It promotes the free secretion of the liver, upon the want of which dyspepsia often depends. I have never salivated a patient severely, and have seen the soreness of the gums disappear whilst the patient was taking pills of cal., al. and rhu. every night. My plan is to administer a full dose—from 12 to 20 grains generally in the beginning, and to follow it with the same article, combined with the dry vegetable purgatives, on the next evening, and to continue the purgatives until the patient is cured. If anything be required to aid the calomel in its action, I prescribe aloes, rhubarb and scammony. According to the view of the disease which I have adopted, the cure depends upon the free action of the liver, and this is maintained by the vegetable cathartics after it has been fully established by the mercurial. It may become necessary occasionally to add a little calomel or blue pill to the other purgatives, should the bilious secretion become languid.

Louisville, 1844.

